# Constructions of solitary wave solutions for huge family of NPDEs with three applications

**DOI:** 10.1371/journal.pone.0318220

**Published:** 2025-01-30

**Authors:** Hanan A. Alkhidhr, Y. Omar

**Affiliations:** 1 Department of Mathematics, College of Science, Qassim University, Buraidah, Saudi Arabia; 2 Department of Mathematics, Faculty of Science, Damietta University, New Damietta, Egypt; COMSATS University Islamabad, PAKISTAN

## Abstract

This study offers closed-form solutions for the frequently utilised families of nonlinear partial differential equations (NPDEs). This form based on the He’s semi-inverse technique. This form can be considered as a box solver for physicists, engineers and mathematicians. This closed form has several advantages, including eliminating complicated calculations and clearly presenting crucial solutions. Three physical applications are provided in order to validate this closed form technique. The theoretical investigation and given results demonstrate that the suggested technique is efficient and appropriate. For appropriate values of the free parameters, some graphs are used to characterize the dynamical changes of the derived solutions. Finally, our methodology may be extended to various equations emerging in several branches of applied science.

## 1 Introduction

Travelling wave solutions of nonlinear partial differential equations (NPDEs) are widely recognised as being crucial for investigating nonlinear wave phenomena [1–4]. Nonlinear phenomena is one of the most fascinating fields for scientists in this modern era. NLPDEs play an important role in describing the physical mechanics of natural occurrences as well as dynamical processes in fluid mechanics, neuroscience, optical fiber communications, chemical engineering, plasma physics, geochemistry, and many other [5–9]. Several strategies for discovering exact solutions to NPDEs have been presented, including sine-cosine method [10], trial solution method [11], F-expansion method [12], RB sub-ODE method [13], $(\frac{G^{\prime }}{G})-$ expansion method [14], Jacobi elliptic function method [15], and so on.

In recent years, significant advancements have been made in the study of nonlinear partial differential equations (NPDEs) and soliton solutions, which are critical for understanding complex physical phenomena. For example, Kai and Yin in [16] investigated the linear structure and soliton molecules of the Sharma-Tasso-Olver-Burgers equation, providing essential insights into nonlinear system behaviors. Similarly, Zhu et al. presented exact soliton solutions to the (2+1) dimensional Chaffee-Infante equation, contributing valuable wave structure analyses applicable to nonlinear dynamics [17]. Moreover, the work by Han et al. on the skidding behavior of cylindrical roller bearings under time-variable conditions offers a comparative perspective on time-variable dynamic systems [18], which are closely related to the models explored in this paper. Additionally, research by Guo and Wang on twisted relative Rota-Baxter operators in Leibniz conformal algebras can enhance the understanding of algebraic approaches in NPDEs [19]. Furthermore, Wu et al. provided a closed-form analytical solution for wave propagation attenuation in periodic structures, which can be beneficial when analyzing similar wave phenomena [20]. Finally, Xi et al. examined high-order interactions in functional brain networks [21], showcasing interdisciplinary applications of dynamic systems relevant to the nonlinear equations studied here.

Consider the NPDEs:

$$\begin{array}{c} \Lambda (Q,Q_{t},Q_{x},Q_{tt},Q_{xx},\ldots .)=0, \end{array}$$ (1.1)

Λ denotes a polynomial of *Q*(*x*, *t*) with partial derivatives. Utilizing the wave transformation with wave speed *v*

$$\begin{array}{c} Q(x,t)=q(\xi ),\;\;\;\;\;\;\;\xi =x-vt \end{array}$$ (1.2)

converts Eq (1.1) into the following ODE:

$$\begin{array}{c} \Gamma (q,q^{\prime },q^{\prime \prime },q^{\prime \prime \prime },\ldots ..)=0, \end{array},$$ (1.3)

while $q^{\prime }\left(\xi \right)=\frac{dq}{d\xi },\;q^{\prime \prime }\left(\xi \right)=\frac{d^{2}q}{d\xi^{2}}$ and so on. Actually, there are huge number of applied models take the following form [22–26]:

$$\begin{array}{c} Lq^{\prime \prime }+Mq^{3}+Nq=0. \end{array}$$ (1.4)

This equation describes a Hamiltonian system with fascinating applications [27]. Because of the significance of Eq (1.4), we propose a closed form solution for the most commonly used NPDEs, using He’s semi-inverse technique [28–30]. This method, in particular, forms the foundation for closed form solution. According to He’s semi-inverse technique [28–30], integrate Eq (1.4) term by term, gives constant(s) of integration that can be chosen zero for simplicity. The trial-function that follows is created using He’s semi-inverse approach:

$$\begin{array}{c} J(q)=\int L\;d\xi , \end{array}$$ (1.5)

L depend on *q* and its derivatives, is the Lagrangian function of Eq (1.4).

In this paper, we look for closed form in the following format:

$$\begin{array}{c} q(\xi )=A\;sech(B\xi ), \end{array}$$ (1.6)

using the Ritz approach, *A* & *B* are constants to be calculated. Substituting Eq (1.6) into Eq (1.5) and making *J* stationary with respect to *A*; *B* produce

$$\begin{array}{c} \frac{\partial J}{\partial A}=0, \end{array}$$ (1.7)

$$\begin{array}{c} \frac{\partial J}{\partial B}=0. \end{array}$$ (1.8)

Solving these equations, gives the values of *A* and *B*. As a result, the solitary wave solution given by Eq (1.6) will be provide.

In this paper we introduce a closed form solution for the huge number of NPDEs arising in applied science. This form based on the He’s semi-inverse technique [28–30]. Engineers, physicists and mathematicians can employ the closed form as a box solver. The suggested approach has several advantages, including eliminating difficult and time-consuming computations and obtaining precise results using physical parameters. This solver is easy, reliable, and durable. We also applied this technique for three models arising in natural science. Specifically, we consider the Phi-4 equation, the new Konno-Oono system and (2+1)-dimensional hyperbolic nonlinear Schrödinger (2D-HNLS) model.

This is how the rest of the manuscript is organized. Section 2 introduces the closed form solution for Eq (1.4). In applied mathematics and physics, this form permits crucial and significant influences. Three applications are provided in Section 3 to ensure that the unified solution is valid. Sec. 4 displays the interpretation of the presented solutions that were given. Additionally, some 2D, 3D charts for the solutions produced for appropriate free parameter values are provided. Section 5 will include the conclusion.

## 2 Closed form of solutions

Using He’s semi-inverse approach [28–30], the variational formulation from Eq (1.4) constructed as follows:

$$\begin{array}{c} J\left(q\right)=\int_{0}^{\infty }(\frac{L}{2}{\left(q^{\prime }\right)}^{2}-\frac{M}{4}q^{4}-\frac{N}{2}q^{2})d\xi . \end{array}$$ (2.1)

Using the Ritz approach, we look for a solitary wave solution in the form (1.6). Substituting Eq (1.6) into Eq (2.1), gives

$$\begin{array}{ccc} J & = & \int_{0}^{\infty }[\frac{L}{2}A^{2}B^{2}\;sech^{2}\left(B\xi \right)\;{\text{tanh}}^{2}\left(B\xi \right)-\frac{M}{4}A^{4}\;sech^{4}\left(B\xi \right)-\frac{N}{2}\;A^{2}\;sech^{2}\left(B\xi \right)]d\xi .\\ & = & \frac{L}{2}A^{2}B^{2}\;\int_{0}^{\infty }sech^{2}\left(B\xi \right)\;{\text{tanh}}^{2}\left(\xi \right)-\frac{M}{4}A^{4}\;\int_{0}^{\infty }sech^{4}\left(B\xi \right)-\frac{N}{2}\;A^{2}\;\int_{0}^{\infty }sech^{2}\left(B\xi \right)d\xi .\\ & = & \frac{L\;A^{2}B}{6}-\frac{M\;A^{4}}{6B}-\frac{N\;A^{2}}{2B}. \end{array}$$ (2.2)

Setting *J* stationary in relation to *A* and *B* results in

$$\begin{array}{c} \frac{\partial J}{\partial A}=\frac{L\;AB}{3}-\frac{2MA^{3}}{3B}-\frac{N\;A}{B}=0. \end{array}$$ (2.3)

$$\begin{array}{c} \frac{\partial J}{\partial B}=\frac{LA^{2}}{6}+\frac{MA^{4}}{6B^{2}}+\frac{N\;A^{2}}{2B^{2}}=0. \end{array}$$ (2.4)

Solving these equations yields:

$$\begin{array}{c} A=\pm \sqrt{\frac{-2N}{M}},\;\;B=\pm \sqrt{\frac{-N}{L}}. \end{array}$$ (2.5)

Therefore, (1.6) solutions have the form

$$\begin{array}{c} q\left(\xi \right)=\pm \sqrt{\frac{-2N}{M}}sech\left(\pm \sqrt{\frac{-N}{L}}\xi \right). \end{array}$$ (2.6)

## 3 Applications

We applied the above closed form solutions for three physical models arising in natural science. The Phi-4 equation, a well-known NPDE in mathematical physics, is the first equation [31]. Numerous natural science fields, such as particle physics and nuclear physics, are studying this model. A variant of the Klein-Gordon (KG) model that is connected to the nonlinear Schrödinger model is the Phi-4 equation. Scientists have employed mathematical and numerical solutions of the Phi-four equation to study several strategies, including sine-cosine technique [32], modified exp(−Ω(*ξ*))-expansion function technique [31], modified simple equation [33], adomian decomposition technique [34], auxiliary equation technique [35], etc. The solutions of this equation may be used to investigate a variety of quantum phenomena, including the ability of matter waves, the fundamental building block of quantum mechanics to govern actuality in the form of waves.

The second model is the new coupled Konno–Oono, which is a coupled integrable dispersionless equations [36–39]. This model is commonly utilised in the magnetic field. This system has received a lot of interest in recent years and has been the topic of several research, such as sine-Gordon expansion approach [36], tanh-function approach & extended tanh-function approach [37], external trial equation approach [38], extended exp function approach [39].

The third model is the 2D-HNLS equation [40–42]. This model describes the kinetics of optical soliton propagation in mono-mode optical fibres. The relevance of investigating the 2D-HNLS equation has prompted many scholars to choose it as a conventional model in their study. Ai-Lin and Ji used the Lie group symmetry approach to identify Lie point symmetries and precise travelling solutions for the 2D-HNLS problem [43]. The optical solitary waves of the 2D-HNLS model were studied by Aliyu et al. using the solitary wave ansatz [40]. Durur et al. used the projected approach to develop singular and periodic wave solutions for the 2D-HNLS model [41].

**Application 1**

The following is the Phi-4 equation [31]:

$$\begin{array}{c} Q_{tt}-Q_{xx}+\alpha^{2}Q+\beta Q^{3}=0, \end{array}$$ (3.1)

where *α*, *β* are real valued constants. The terms *Q*_*tt*_ and *Q*_*xx*_ represent the effect of dissipation, whereas the term *Q*^3^ denotes the nonlinearity effect. Utilizing the wave transformation

$$\begin{array}{c} Q(x,t)=q(\xi ),\;\;\;\;\;\;\xi =x-vt, \end{array}$$ (3.2)

Eq (3.1) goes to

$$\begin{array}{c} \left(v^{2}-1\right)q^{\prime \prime }+\beta q^{3}+\alpha^{2}q=0\;. \end{array}$$ (3.3)

The closed form solution of this equation is

$$\begin{array}{c} q_{1,2}\left(\xi \right)=\pm \sqrt{\frac{-2\alpha^{2}}{\beta }}\;\text{sech}(\pm \sqrt{\frac{\alpha^{2}}{\left(1-v^{2}\right)}}\xi ),\;\;\beta <0,-1<v<1. \end{array}$$ (3.4)

Consequently, the solutions of Eq (3.1) are

$$\begin{array}{c} Q_{1,2}\left(x,t\right)=\pm \sqrt{\frac{-2\alpha^{2}}{\beta }}\;\text{sech}(\pm \sqrt{\frac{\alpha^{2}}{\left(1-v^{2}\right)}}\left(x-vt\right)),\;\;\beta <0,-1<v<1. \end{array}$$ (3.5)

**Application 2**

The new Konno-Oono system is given by [36–39]:

$$\begin{array}{c} \begin{array}{c} \psi_{xt}-2\psi \;\phi =0,\\ \phi_{t}+2\psi \psi_{x}=0. \end{array} \end{array}$$ (3.6)

Using the transformation

$$\begin{array}{c} \psi (x,t)=\psi (\xi ),\;\;\phi (x,t)=\phi (\xi ),\;\;\xi =x+\lambda \;t, \end{array}$$ (3.7)

λ is the wave speed. Plugging (3.7) into (3.6), gives

$$\begin{array}{c} \lambda \;\psi^{\prime \prime }-2\psi \phi =0, \end{array}$$ (3.8)

$$\begin{array}{c} \lambda \;\phi^{\prime }+2\psi \psi^{\prime }=0. \end{array}$$ (3.9)

Integrating Eq (3.9) with respect to *ξ*, gives

$$\begin{array}{c} \phi =\frac{-1}{\lambda }\left(\psi^{2}+K\right), \end{array}$$ (3.10)

*K* is an integral constant. Putting Eq (3.10) into Eq (3.8), gives

$$\begin{array}{c} \lambda^{2}\;\psi^{\prime \prime }+2\psi^{3}+2K\psi =0. \end{array}$$ (3.11)

The closed form solution of this equation is

$$\begin{array}{c} \psi_{1,2}\left(\xi \right)=\pm \sqrt{-2K}sech\left(\pm \frac{\sqrt{-2K}}{\lambda }\;\xi \right),\;K<0,\lambda \neq 0. \end{array}$$ (3.12)

Thus, the solutions for Eq (3.6) are

$$\begin{array}{c} \psi_{1,2}\left(x,t\right)=\pm \sqrt{-2K}sech\left(\pm \frac{\sqrt{-2K}}{\lambda }\left(x+\lambda \;t\right)\right),\;K<0,\lambda \neq 0. \end{array}$$ (3.13)

$$\begin{array}{c} \phi_{1,2}\left(x,t\right)=\frac{K}{\lambda }\left(2sech^{2}\left(\pm \frac{\sqrt{-2K}}{\lambda }\left(x+\lambda \;t\right)\right)-1\right),\;K<0,\lambda \neq 0. \end{array}$$ (3.14)

**Application 3**

The (2+1)-dimensional hyperbolic nonlinear Schrödinger equation given by [40–42]:

$$\begin{array}{c} i\phi_{y}+\frac{1}{2}\left(\phi_{xx}-\phi_{tt}\right)+|\phi {|}^{2}\phi =0. \end{array}$$ (3.15)

where, *ϕ*(*x*, *y*, *t*) describes the complex wave, *x*, *y* denote the position variables and *t* denotes the time variable. Using the transformation

$$\begin{array}{c} \phi \left(x,y,t\right)=\Phi \left(\xi \right)e^{i(x+\alpha_{2}y+\beta_{2}t)},\;\;\;\xi =x+\alpha_{1}y+\beta_{1}t,\; \end{array}$$ (3.16)

*β*_1_ and *β*_2_ demonstrate speed & frequency of the solitary wave. Putting Eq (3.16) into Eq (3.15) and differentiating the real part results in

$$\begin{array}{c} \left(1-\beta_{1}^{2}\right)\Phi^{\prime \prime }+2\;\Phi^{3}+\left(\beta_{2}^{2}-2\alpha_{2}-1\right)\;\Phi =0\;, \end{array}$$ (3.17)

and imaginary part gives *α*_1_ = *β*_1_*β*_2_ − 1. The closed form solution Eq (3.17) is

$$\begin{array}{c} \Phi_{1,2}\left(\xi \right)=\pm \sqrt{1+2\alpha_{2}-\beta_{2}^{2}}\;sech\left(\pm \sqrt{\frac{1+2\alpha_{2}-\beta_{2}^{2}}{1-\beta_{1}^{2}}}\;\xi \right),1+2\alpha_{2}-\beta_{2}^{2}>0,1-\beta_{1}^{2}>0. \end{array}$$ (3.18)

Thus, the solutions for Eq (3.15) are

$$\begin{array}{c} \phi_{1,2}\left(x,y,t\right)=\pm \sqrt{1+2\alpha_{2}-\beta_{2}^{2}}\;e^{i(x+\alpha_{2}y+\beta_{2}t)}\;sech(\pm \sqrt{\frac{1+2\alpha_{2}-\beta_{2}^{2}}{1-\beta_{1}^{2}}}\left(x+\alpha_{1}y+\beta_{1}t\right)), \end{array}$$ (3.19)

$1+2\alpha_{2}-\beta_{2}^{2}>0,1-\beta_{1}^{2}>0$.

## 4 Results and discussion

In this paper we constructed closed form of solutions for a widely used of NPDEs using He’s semi-inverse technique. Specifically, we provided a box solver for engineers, mathematicians and physicists. This form of solutions, known as a hyperbolic secant solutions, can be occur in the profile of a laminar jet [44]. This closed form does away with time-consuming and challenging calculations. We can repeat the same strategy of closed from in order to produce another closed form, such as *Acsch*(*Bξ*).

**Fig 1:**
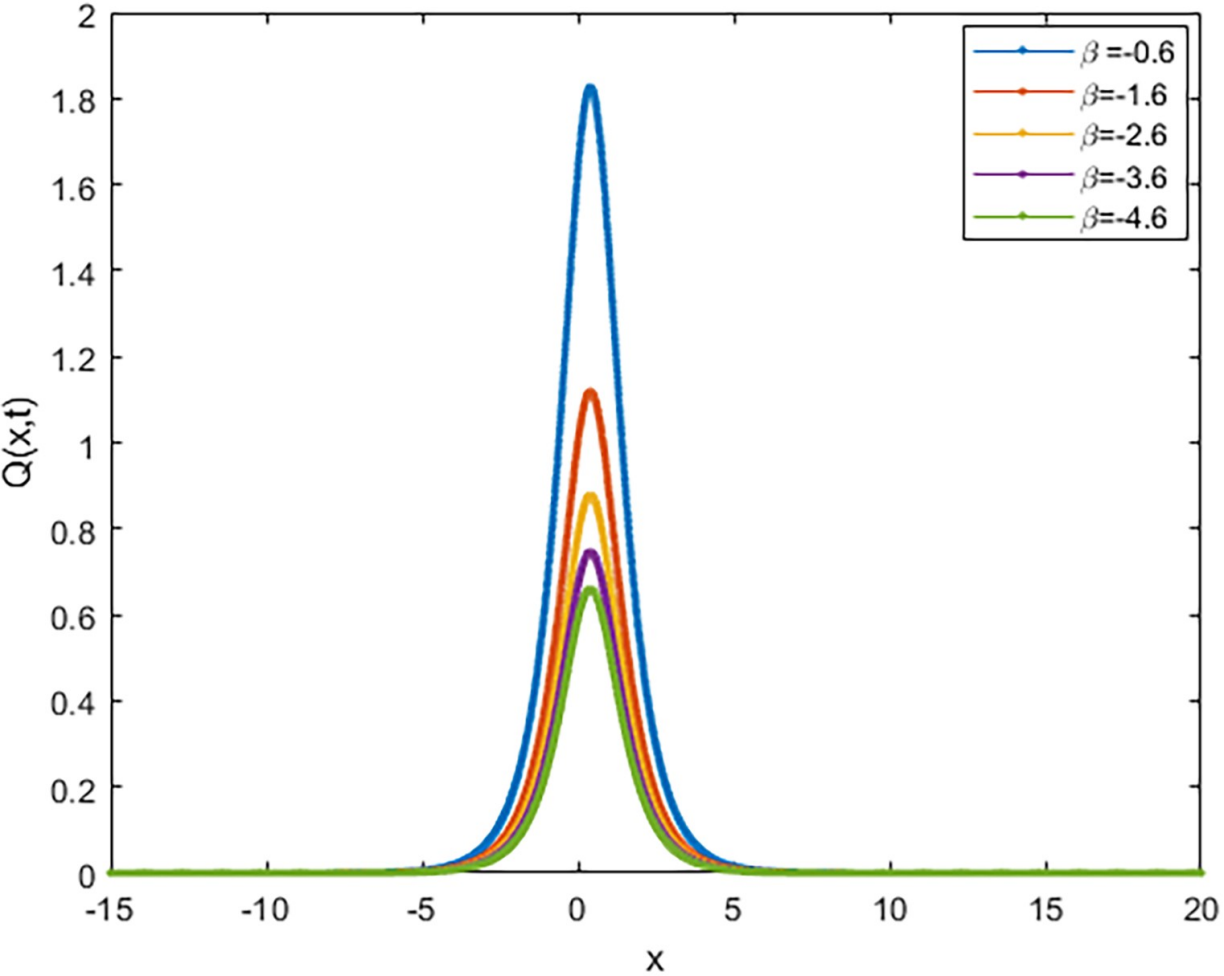
2D graph of soliton wave solution (3.5) for distinct values of *β*.

**Fig 2:**
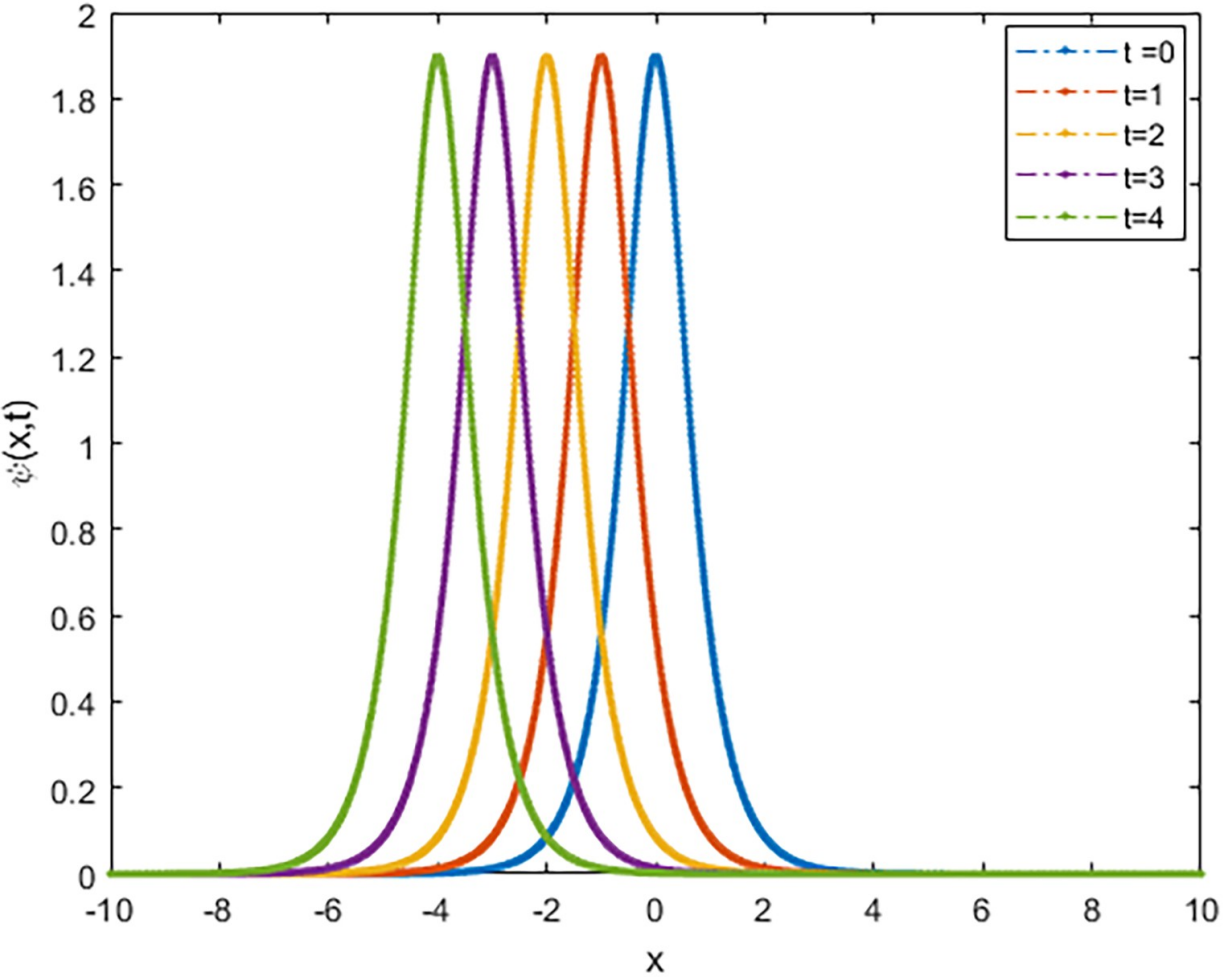
2D graph of soliton wave solution (3.13) for distinct values of *t*.

**Fig 3:**
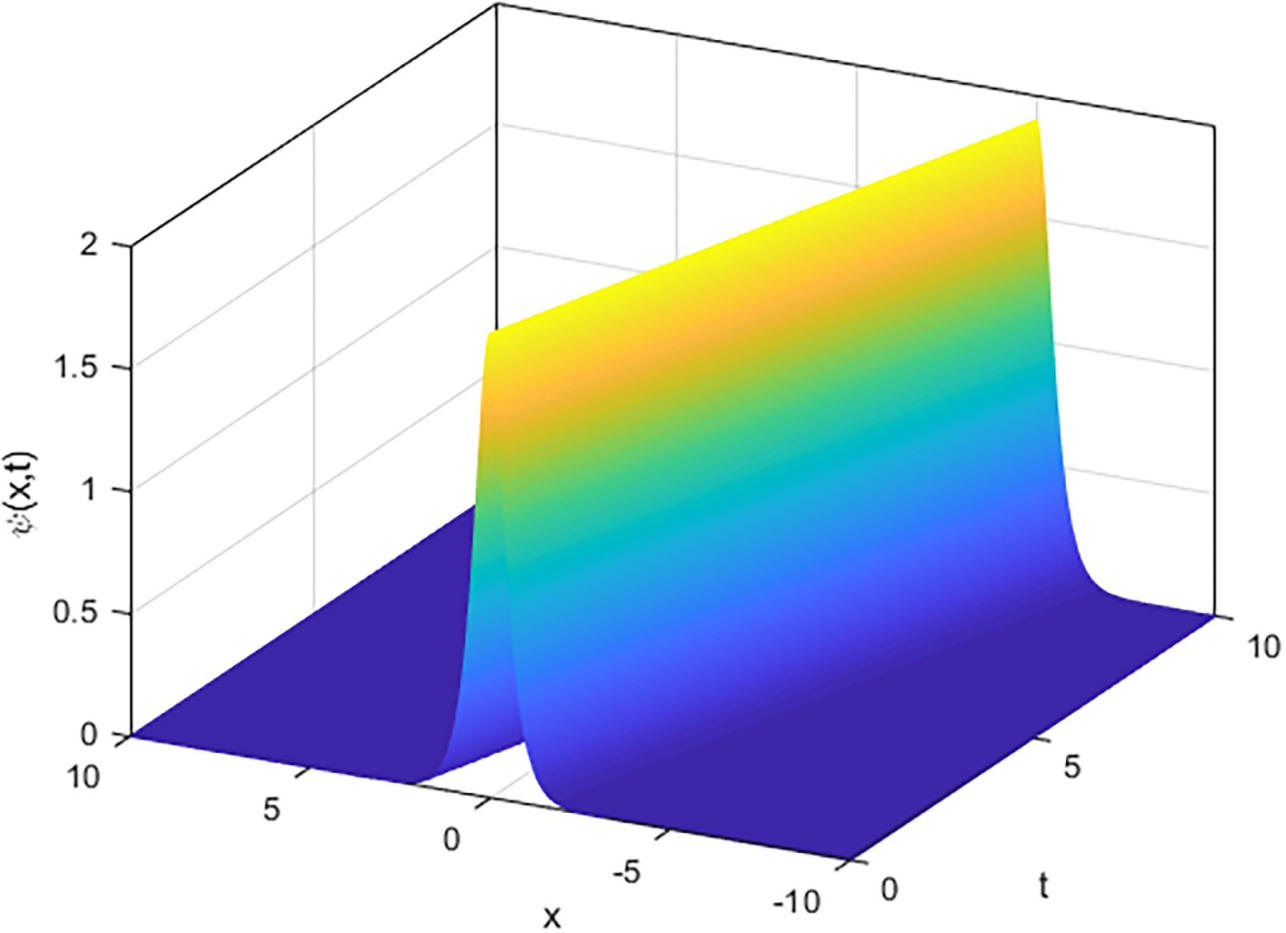
3D graph of soliton wave solution (3.13).

**Fig 4:**
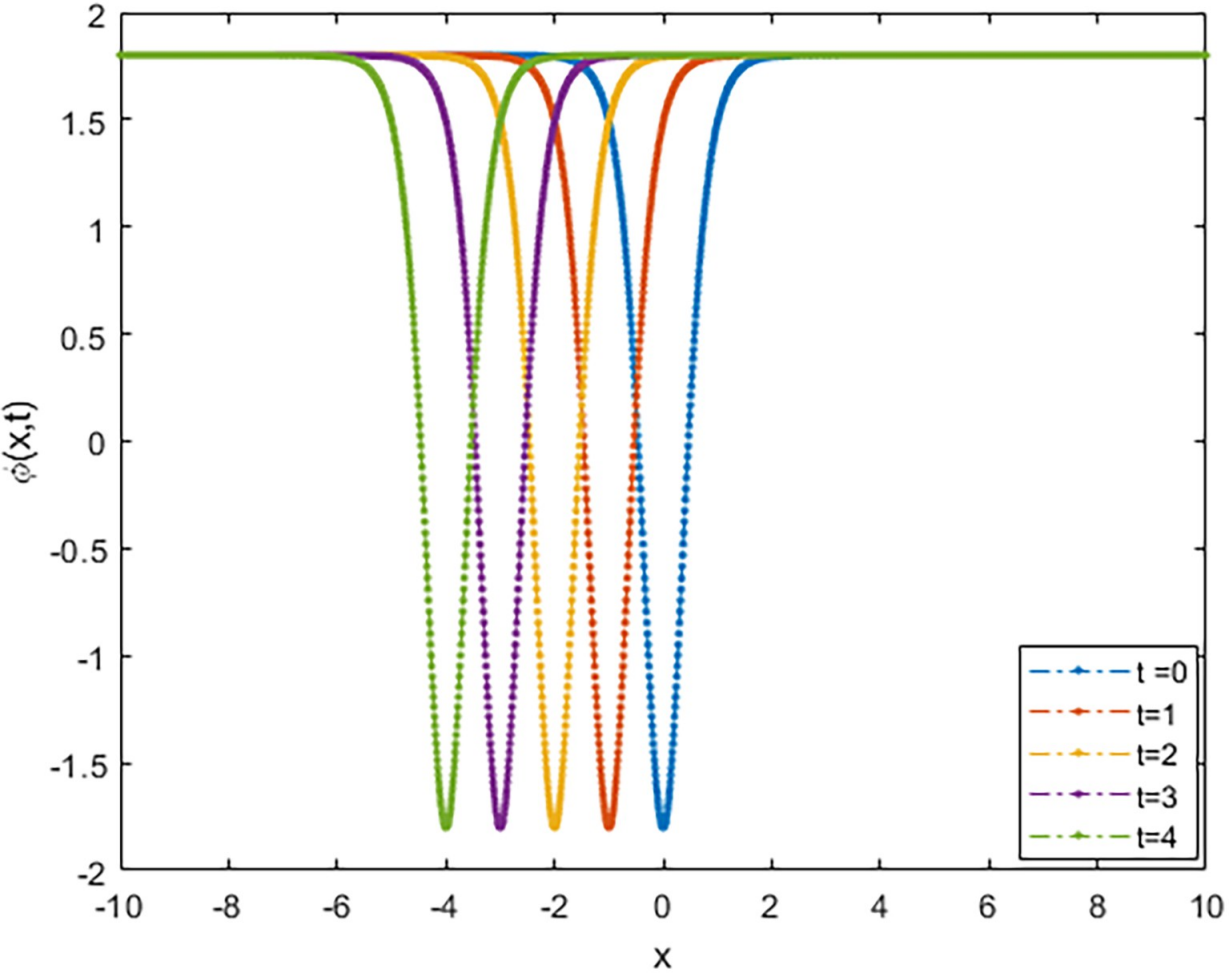
2D graph of soliton wave solution (3.14).

**Fig 5:**
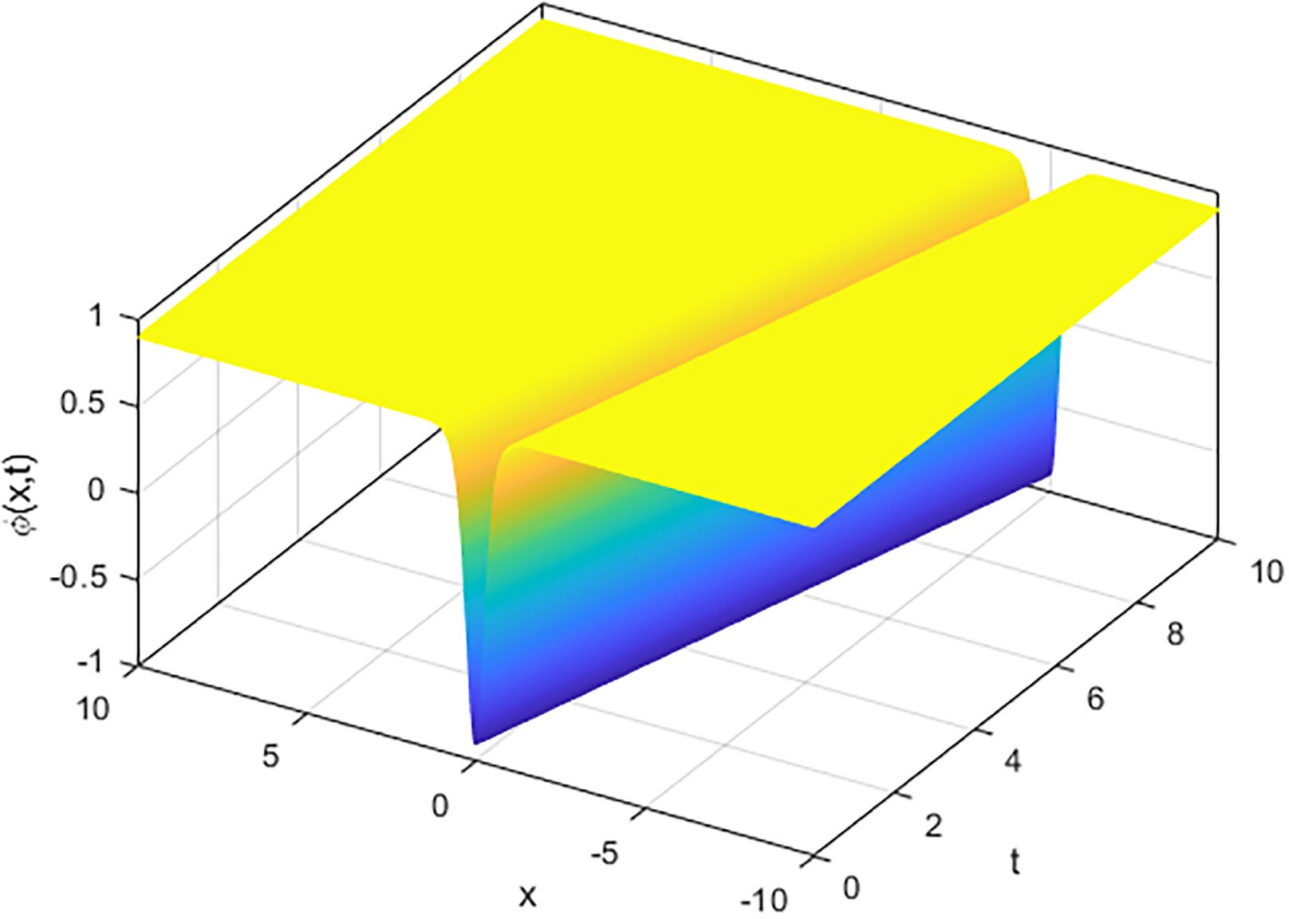
3D graph of soliton wave solution (3.14).

**Fig 6:**
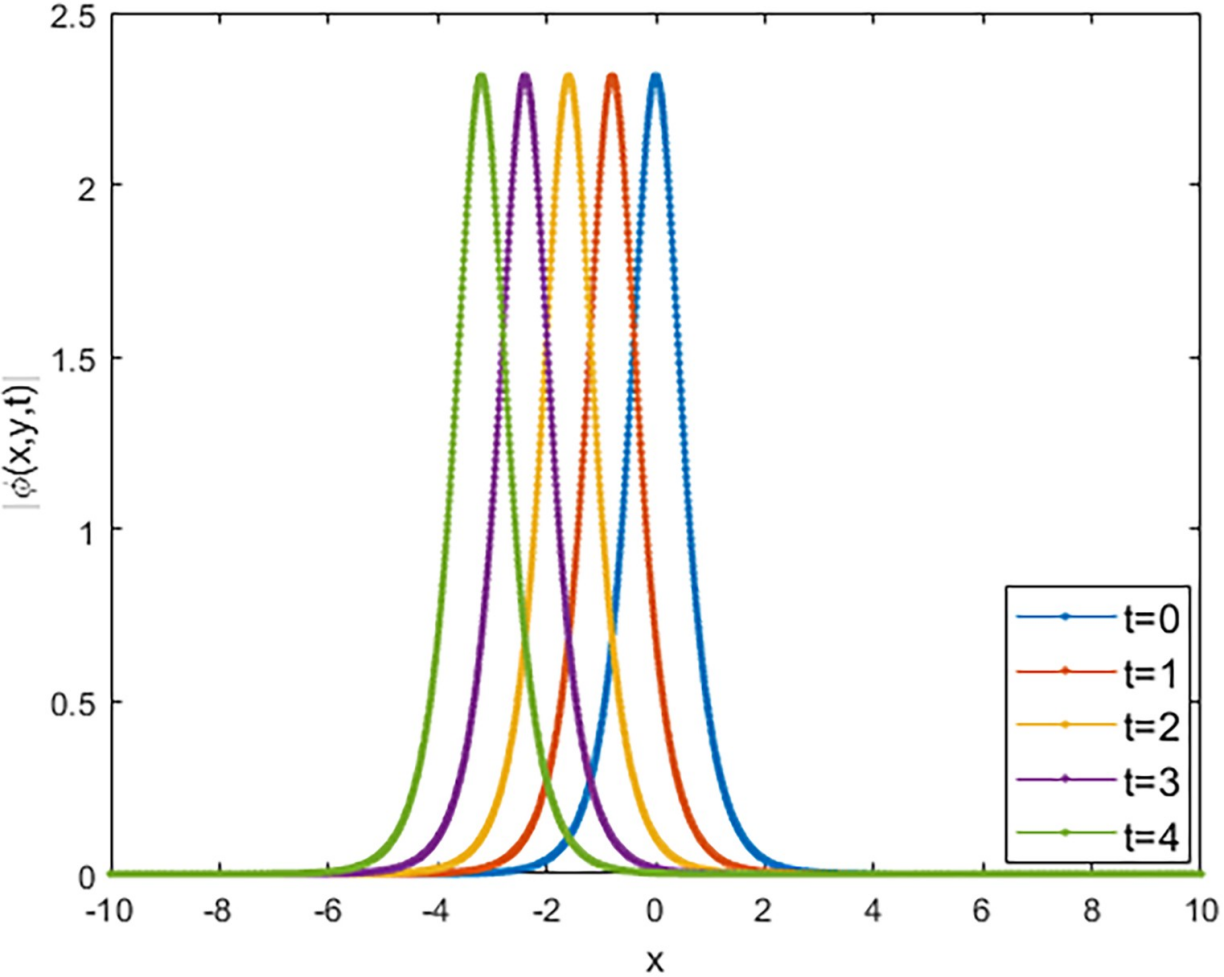
2D graph of soliton wave solution (3.19).

We applied the closed form of solutions to three physical systems arising in new physics and applied science. Namely, we introduced vital solutions for Phi-4 equation, which of great important in nuclear physics, particle physics. We gave critical solutions for the new Konno-Oono system, which is extremely important in magnetic fields. We also obtained key solutions for the 2D-HNLS equation, which are critical in mono-mode optical fibers. In applied sciences, these solutions provide wave images that characterise complicated phenomena. The fact that this solver can be used to solve a wide variety of models of nonlinear fractional differential equations is one of its key features [45, 46]. Fig 1 shows that decreasing the parameter *β* decreases the amplitude of the soliton wave. We also illustrated the behaviour of solutions for model (3.6) through 2D and 3D Figs 2–5. The graphical representation of ∣*ϕ*_1_(*x*, *y*, *t*)∣ demonstrates the soliton wave of solution (3.19) as shown in Fig 6.

The results reveal that the proposed technique is successful and can create a significant number of wave solutions for NPDEs, which will be valuable in the investigation of solitary theory in applied science. For example, this solver can be easily applied for the modified Korteweg-de Vries (mKdV) equation, chiral nonlinear Schrödinger equation, nonlinear Maccari’s models, Heisenberg ferromagnetic spin chain equation and other more. One of the primary aspects of this solver is its ability to handle a wide range of nonlinear fractional differential equation models. The limitation of the proposed solver is that, it considers only the NPDEs which convert to from (Fig 1). So we will surely try to find another closed form for other models of NPDEs, which are not convert to this form in the forthcoming paper.

## 5 Conclusions

We have created closed form solutions in this work using the He’s semi-inverse method. We implemented this form for solving three models of nonlinear partial differential equations. Namely, we solved the Phi-4 equation, the new Konno-Oono system and the 2D-HNLS equation. The presented results are expected to help understand several physical phenomena in various nonlinear mathematical models of physics. Many scientists will solve several other complex models that arise in the applied sciences using the closed form as a box solver.

## Supporting information

S1 Dataset (PDF)
