# Supplementary material for: Constructions of solitary wave solutions for huge family of NPDEs with three applications
Source: PLoS One. 2025 Jan 30;20(1):e0318220. doi: 10.1371/journal.pone.0318220 (PMC11781697; doi:10.1371/journal.pone.0318220)
Supplement: S1 Dataset — (PDF) [file pone.0318220.s001.pdf]

Figures	Solution	α	β	*v*	*t*	x
Figure 1	$Q_1(x, t)$	1	- 0.6,-1.6,-2.6,-3.6,-4.6	0.4	1	[-15 20]

Figures	Solution	K	λ	*t*	x
Figure 2	$\Psi_1(x, t)$	-1.8	1	0,1,2,3,4	[-10 10]
Figure 3	$\Psi_1(x, t)$	-1.8	2	[0 10]	[-10 10]
Figure 4	$\phi_1(x, t)$	-1.8	1	0,1,2,3,4	[-10 10]
Figure 5	$\phi_1(x, t)$	-1.8	2	[0 10]	[-10 10]

Figures	Solution	α_1	α_2	β_1	β_2	*t*	*y*	x
Figure 6	$\phi_1(x,y, t)$	0.5	2.5	0.2	0.8	0, 1, 2, 3, 4	0	[-10 10]
